# Corrosion mechanism and kinetics of Al-Zn coating deposited by arc thermal spraying process in saline solution at prolong exposure periods

**DOI:** 10.1038/s41598-019-39943-3

**Published:** 2019-03-04

**Authors:** Han-Seung Lee, Jitendra Kumar Singh, Mohamed A. Ismail, Chinmoy Bhattacharya, Asiful H. Seikh, Nabeel Alharthi, Raja Rizwan Hussain

**Affiliations:** 1 Department of Architectural Engineering, Hanyang University, 1271 Sa 3-dong, Sangrok-gu, Ansan 15588 Korea; 2 Department of Civil Engineering, Miami College of Henan University, Jinming Avenue No.1, Kaifeng, Henan 475001 China; 3 Department of Chemistry, Indian Institute of Engineering Science and Technology (IIEST), Shibpur, Howrah, 711 103 West Bengal India; 4 Centre of Excellence for Research in Engineering Materials, King Saud University, P.O. Box 800, Riyadh, 11421 Saudi Arabia; 5 Mechanical Engineering Department, King Saud University, P.O. Box 800, Riyadh, 11421 Saudi Arabia; 6 College of Engineering, Department of Civil Engineering, King Saud University, P.O. Box - 800, Riyadh, 11421 Saudi Arabia

## Abstract

Steel structures significantly degrades owing to corrosion especially in coastal and industrial areas where significant amounts of aggressive ions are present. Therefore, anodic metals such as Al and Zn are used to protect steel. In the present study, we provide insights for the corrosion mechanism and kinetics of Al-Zn pseudo alloy coating deposited on mild steel plate via an arc thermal spraying process in 3.5 wt.% NaCl solution in terms of its improved corrosion resistance properties at prolonged exposure durations. Electrochemical studies including open circuit potential (OCP) and electrochemical impedance spectroscopy (EIS) on the deposited coating at longer exposure durations revealed enhanced corrosion resistance properties while the morphology of corrosion products through field emission-scanning electron microscopy (FE-SEM) indicated their compactness and adherence. Furthermore, atomic force microscopy (AFM) confirmed reduced roughness when compared with that of unexposed coating. Additionally, X-ray diffraction (XRD) and Raman spectroscopy results confirmed the formation of protective, adherent, and sparingly soluble Simonkolleite (Zn_5_(OH)_8_Cl_2_.H_2_O) after 55 d of exposure in 3.5 wt.% NaCl solution. A schematic is proposed that explains the corrosion process of Al–Zn pseudo alloy coating in 3.5 wt.% NaCl solution from the deposition of coating and initiation of corrosion to longer exposure durations.

## Introduction

Corrosion of steel structures in an aggressive environment is a worldwide problem and is unavoidable. Among different environments, the sea water environment is considered as the most aggressive towards corrosion degradation. Hence, the equipment or its components used in marine environment suffers from severe corrosion degradation, and it is essential to control the corrosion rate by applying an imperative protective system^1^.

The most widely used protective system involves the deposition of sacrificial metals such as barrier types of coating on a steel substrate. To date, cadmium, zinc, zinc alloys, Zn-Co-Fe, and zinc-resin hybrid coating^2–8^ and chromium- or nickel-based^9,10^ systems are most commonly used to protect steel structures from corrosion. Furthermore, hot dip galvanized coating and heavy duty paint that contain zinc and zinc rich primer with epoxide and fluoride resin are also used to protect steel structures in aggressive environments^11–13^. Among the protective systems, Zn and a combination of Zn with Al and Mg among others is most widely examined. For example, Y. Li (2001) reported that the addition of Al in Zn increases the corrosion resistance properties of galvanized coating after two years of exposure in a seawater environment due to its optimum combination that is resistive to uniform and pitting corrosion^14^. Gulec *et al*.^15^ examined the effect of Al addition in Zn coating on corrosion characteristic of steel exposed to accelerated condition and indicated pronounced corrosion resistance of the Zn/15Al coating^15^. In the Al-Zn coating, Zn provides cathodic protection while Al provides erosion resistance^16,17^.

Although the hot dip galvanized coating of Al-Zn system provides significant corrosion resistance, the process is neither environmental friendly nor conventional in terms of depositing the Al-Zn coating on corroded or big infrastructure on-site in addition to several negative impacts on humans^18–20^. Therefore, it is imperative to replace aforementioned coating processes via other conventional coating methods. Among different coating processes, thermal spray coating appears promising to protect steel components from corrosion degradation.

Different thermal spray processes including high-velocity oxy-fuel, plasma, and arc thermal spray are widely used globally for metals, ceramic, and plastic coating to provide wear resistance, heat insulation, and corrosion resistance to the metals^21^. Among the thermal spray processes, the arc thermal spray coating process is most suitable and exhibits the advantage of on-site coating on big-infrastructures commonly used in industry for several decades^22,23^, thereby leading to the commercialization of the process. The process exhibits advantage over other coating methods since it is cost effective, highly efficient, and suitable to coat engineering parts with sacrificial metals, such as Zn, Al, and their alloys, to enhance the corrosion resistance properties of steel at longer durations of exposure in aggressive environments^24–26^. The process exhibits a high deposition rate of the metallic coating on a substrate, and thus is attractive in terms of industrial applications^27^.

Despite the ease of the process and the capability of on-site coating that leads to good corrosion resistance, the process suffers from issues of porosity in the coating, and this is an inherent property of arc thermal spray process. Lee *et al*. investigated the corrosion characteristic of Al coating deposited via the arc thermal spray process in saline as well as simulated weathering conditions and indicated that corrosion resistance properties increased significantly at longer duration of exposure due to the formation of a protective film that filled the pores/defects of Al coating^28,29^. Another alternative to fill the pores/defects of Al coating involves using post treatment that enhances corrosion resistance properties in artificial ocean water solution at prolonged exposure periods^30,31^. The alloying of Zn and Mg plays an important role in filling out the pores and enhancing the corrosion resistance properties of Al coating deposited via the arc thermal spraying process in simulated weathering conditions^32^. The arc thermal spraying process is used to protect waste water reservoirs from acidic corrosion by using Ti and stainless steel coating on concrete^33,34^. However, the arc thermal spraying process is limited in the case of Al and Zn coatings due to their sacrificial nature with respect to steel. It is predicted that Al and Zn coatings protect steel by dissolving the oxides/coatings and preferentially fill the pores/defects of coating by corrosion products^24^. This increases the corrosion resistance of the coating. However, the exact corrosion mechanism in such coating is neither well examined nor well understood.

The aforementioned literature review indicates that a gap exists in terms of evidences and systematic studies with respect to the protection mechanism of Al-Zn coating on a steel substrate deposited via the arc thermal spray process. Therefore, to the best of the authors’ knowledge, this is the first attempt to systematically examine the kinetics and mechanism of the corrosion of Al-Zn pseudo alloy coating deposited via the arc thermal spraying process in 3.5 wt.% NaCl solution (sea water environment) at longer durations of exposure with different analytical techniques. Most extant studies involved a shorter duration, most included an open atmosphere, and many did not include a simulated condition. The present study provides insights of the corrosion mechanism and kinetics of the aforementioned coating. Additionally, a schematic diagram is used to explain the corrosion mechanism and kinetics of Al-Zn coating.

## Results and Discussion

### Coating thickness and adhesion measurement

The average coating thickness was measured at different locations, and it was approximately 100 µm (±5 µm).

In the present study, the pull off experiment of coating was performed for four samples and their average value was 3.91 ± 0.09 MPa. For the measurement of the adhesion test, the coating surface area was 1.6 × 10^−3^ m^2^, and this exceeds that recommended in KS F4716^35^.

### Morphology of coating

**Figure 1:**
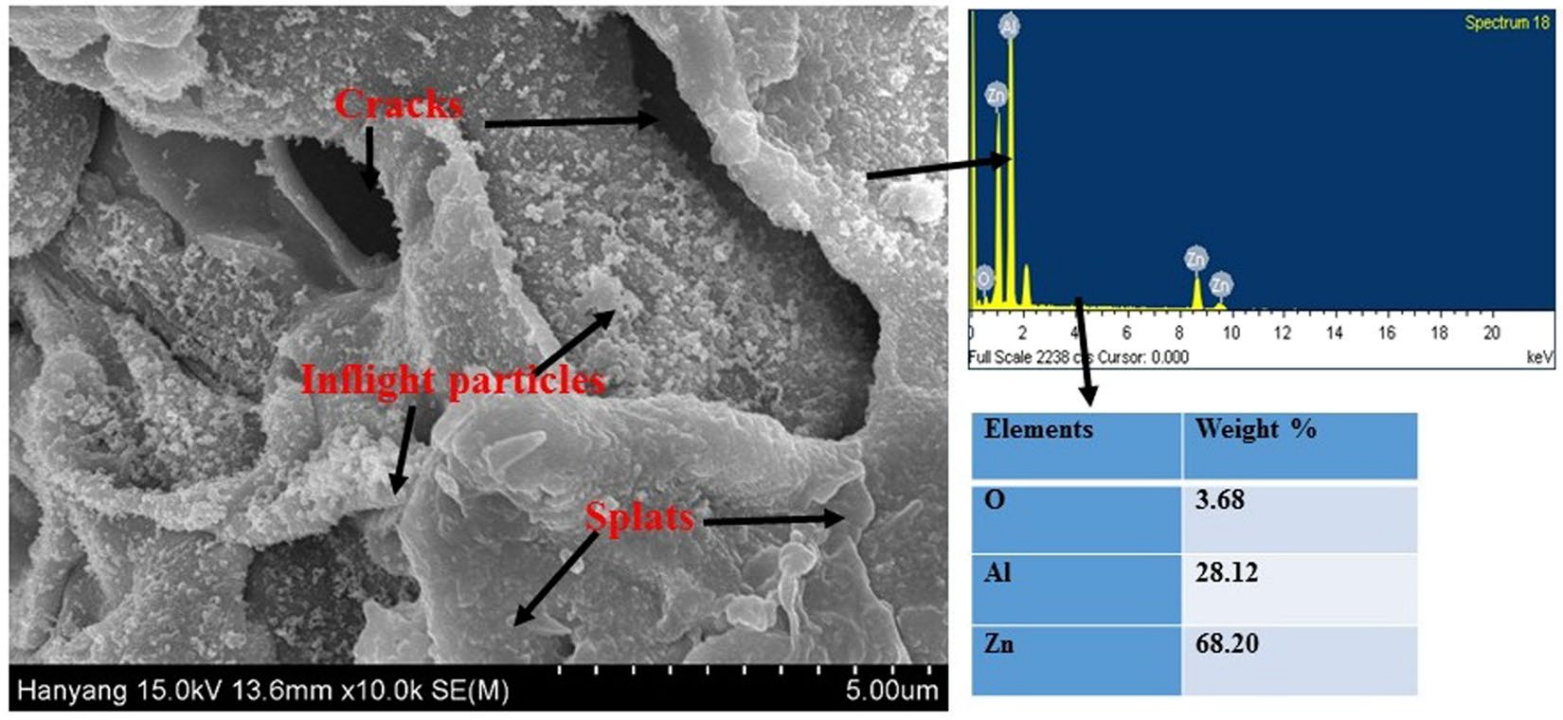
FE-SEM image and EDS of Al-Zn coating.

Figure 1 shows the FE-SEM micrographs of the coating that reveals the coating morphology. The top surface of coating exhibits a splat zone, plate like morphology with cracking, pores, and inflight particles. The inflight particles are extremely small and partially oxidized suspended particles in atmosphere during spraying that can subsequently deposit on the top surface of coating. Macro pores are seen beneath the splat/plate particles in Fig. 1. The splat zones and cracks arise due to sudden cooling of melted metal and continuous layer by layer deposition of coating, respectively. The white deposition over the top surface of coating can be due to inflight particles that come from atmosphere during the spraying process.

The pores/defects formation is an inherent property of thermal spray process^36^, and thus are unavoidable. Feed stoke value, pressure, and distance from target substrate are the governing parameters towards the defect formation and its control. Furthermore, the alteration of the parameters changes the morphology of the coating. However, the diameter of the twin wires feedstock is only 1.6 mm, and this leads to significant defects on the coating surface.

The EDS analysis result is denoted by arrows on the right side of Fig. 1. The spectrum of EDS (right side of Fig. 1) mainly consists of Al, O, and Zn. The basic composition of coating are Al and Zn although O (3.68 wt.%) is introduced from atmosphere or partial oxidation of metals during the deposition of coating. The Al and Zn contents in the coating are 28.12 and 68.20 wt.%, respectively.

### Phase identification of coating

**Figure 2:**
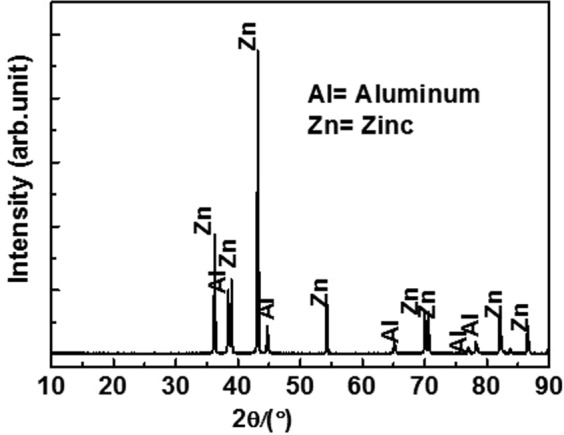
XRD of Al-Zn coating.

It is extremely important to determine the nature of coating by analyzing the phases present on top surface of coating that directly encounter an aggressive environment. Therefore, the XRD analysis was performed, and its result is shown in Fig. 2. The phases present on top surface of coating that pertain to Al and Zn are well matched with those corresponding to the joint committee on powder diffraction standards (JCPDS) number 85–1327 and 87–0713, respectively. In addition to the phases, no other planes are observed in the XRD pattern. However, the presence of O according to EDS result is not detected in the XRD. This is potentially because the contents of other oxides of Al/Zn are extremely low and are beyond the detection limit of the XRD instrument^37–39^. The arc thermal spraying process coating forms mechanical bonds, and thus Al and Zn does not react with each other. Therefore, no intermetallic phase is observed. However, there is a possibility for the formation of an intermetallic phase of Al_0.403_Zn_0.597_ although it overlaps with Al^24^. Thus, it is not observed in present study. Furthermore, the volume fraction (*V*_*f*_) of Al and Zn on coating surface is calculated via integrated surface area calculation^40–43^ as follows:

1 $${V}_{{f}({Al})}=\frac{{A}_{Al}}{{A}_{Al}+{A}_{Zn}}$$

2 $${V}_{{f}({Zn})}=\frac{{A}_{Zn}}{{A}_{Al}+{A}_{Zn}}$$

where *V*_*f(Al)*_ and *V*_*f(Zn)*_ denote the volume fractions, and *A*_*Al*_ and *A*_*Zn*_ denote the total integrated surface area of Al and Zn, respectively. Based on the above calculation, the *V*_*f(Al)*_ and *V*_*f(Zn)*_ are 31.50% and 68.50%, respectively. We also use surface using matrix-flushing theory^44^ to quantify the amount of phases present on coating to corroborate the above calculated results. By using the theory, 29.05% Al and 67.27% Zn (excluding 3.68% oxygen) are obtained and are almost identical to those calculated via the integrated surface area calculation method. Thus, the XRD analysis results indicate that the deposition of Al and Zn metal coatings by the twin wires system forms a pseudo alloy as opposed to a pure metallic coating.

Next, we present the electrochemical corrosion results of the coating performed in a stimulated sea water environment, i.e., 3.5 wt.% NaCl solution.

### Electrochemical studies of coating in 3.5 wt.% NaCl solution

#### Potentiodynamic polarization measurements

**Figure 3:**
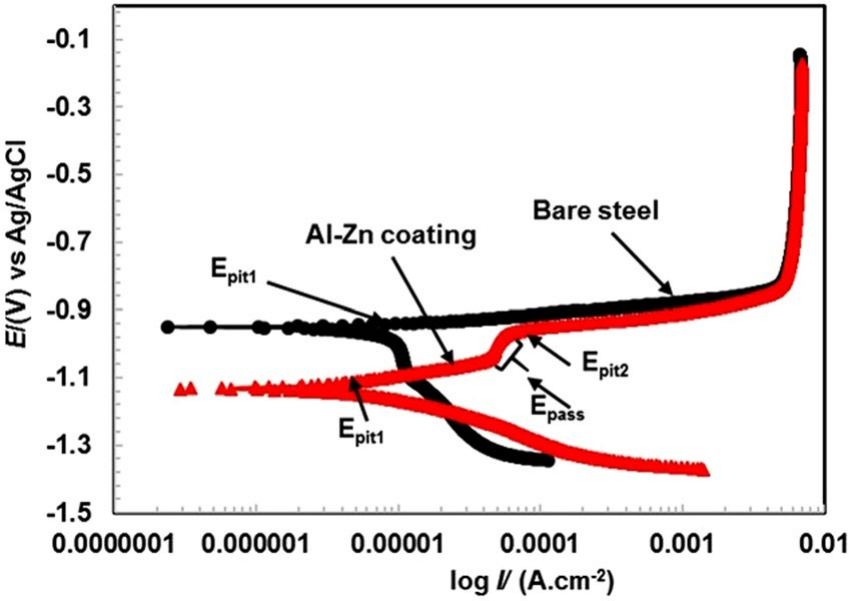
Potentiodynamic plots of Al-Zn coating and bare steel in 3.5wt.% NaCl solution after 1 h of exposure.

The potentiodynamic polarization plots of Al-Zn pseudo alloy coating when compared to bare steel after stabilization of potential for 1 h of exposure in 3.5 wt.% NaCl solution are shown in Fig. 3. The following features are observed from the potentiodynamic polarization curves; (1) The Al-Zn coated sample is cathodically more polarized than the bare one; and (2) both the coated and bare sample exhibit a pitting tendency. However, in the coated sample, two pit regions are observed at −1.110 and −0.966 V vs Ag/AgCl while only one pitting region at 0.940 V vs Ag/AgCl is observed in the bare sample; (3) a passive region is observed in the case of the coated sample, and (4) E^0^_corr_ of the bare sample is more positive than that of the coated sample. Next, we present an explanation of the aforementioned observations and finally list descriptions of electrochemical parameters such as *CR* and *I*_*corr*_ data.

Enhanced cathodic polarization of the coated sample when compared to that of the virgin sample is attributed to the oxygen reduction reaction. Given the reaction, OH^−^ ion enrichment occurs in solution and increases the local pH. In the alkaline pH, the coating suffers local dissolution at pit sites where the pH in the pit decreases and hydrogen gas evolution (bubble) at the coating surface is observed by the naked eye^45^. The result confirms that Al-Zn is under an active dissolution that provides cathodic protection to the steel surface.

Simultaneously, during stabilization of potential, the coating reacts with aggressive electrolyte and forms a few corrosion products/oxides. Therefore, the cathodic current density of coated substrate exceeds that of bare steel. Corrosion products/oxides can be deposited on the active site of electrode surface and reduce the dissolution of coating^45^.

During anodic scanning, the pitting tendency was observed in both samples (i.e., coated and bare steel) although bare steel exhibits a sharp increment in current density (Fig. 3) that is attributed to the susceptibility of surface for corrosion in 3.5 wt.% NaCl solution^46^. Conversely, the coated sample exhibits two pitting potentials (*E*_*pit*_) at different regions as denoted by the arrows in Fig. 3. The *E*_*pit1*_ of bare and coated sample are observed at −0.940 V and −1.110 V vs. Ag/AgCl, respectively. This observation is attributed to the attack of chloride ions on the surface that initiates corrosion phenomena in both cases. It should be noted that the coated sample exhibits active potential than the bare sample either due to the occurrence of mixed potential or due to the more anodic nature of Al and Zn. The mixed potential is caused by the porosity of coating that allows the penetration of aggressive ions towards substrate and induces the faster corrosion reaction^47,48^. The *E*_*pit2*_ on coated sample at −0.966 V vs. Ag/AgCl is due to the removal of loosely bound oxides that form during the initial period of exposure.

An interesting observation is shown in Fig. 3 where the coated substrate exhibits passive region (*E*_*pass*_) either due to deposition of thin corrosion product layer that is generated during initial period of exposure or formation of new phase of oxides that are deposited on pits. Thereafter, it exhibits pitting properties as shown in Fig. 3, and this is potentially due to the attack of Cl^−^ ions on the passive film and formation of a non-protective zincate (ZnO/ZnCl_2_) film for extremely short duration of exposure although it forms different Al and Zn corrosion products containing Cl^−^/OH^−^ ions, such as Al, Zn, and Zn_5_(OH)_8_Cl_2_.H_2_O compounds, at longer durations of exposure^49,50^.

During the initial period of exposure in a saline environment, both samples corrode and form corrosion products exhibiting mass transfer resistance at 4.86 µA/cm^2^ from −0.840 to −0.140 V vs. Ag/AgCl at the applied potential and cause limiting current density due to the corrosion of both samples.

The electrochemical parameters are extracted after extrapolating potentiodynamic polarization plots in Tafel regions, and the results are shown in Table 1. The corrosion rate (µm/year) is calculated by inputting the electrochemical data obtained from potentiodynamic polarization curves in the following equation^51^:

3 $${\rm{Corrosion}}\,{\rm{rate}}\,(\mu {\rm{m}}.{{\rm{y}}}^{-1})=\frac{\,3.27\times {I}_{corr}\times E.\,W.}{d}$$

where *I*_*corr*_ (µA/cm^2^) is obtained by dividing the total surface area of the working electrode in the corrosion current (µA), *E*.*W*. denotes the equivalent weight (g/mol), and *d* denotes the density (g/cm^3^) of metals.

**Table 1:** Electrochemical parameters of potentiodynamic polarization after extrapolation of curve in Tafel regions in 3.5 wt.% NaCl solution for 1 h of exposure.

Sample ID	Electrochemical parameters		
	*E*_*corr*_ (V) vs. Ag/AgCl *I*_*corr*_ (µA/cm^2^)		Corrosion rate (µm/year)
Bare steel	−0.950	20.10	233.17
Al-Zn	−1.123	7.19	120.40

The corrosion potential (${{E}}_{{corr}}^{{o}}$) of Al-Zn coating is −1.123 V vs. Ag/AgCl while bare steel exhibits −0.950 V vs. Ag/AgCl. The more active potential of the coated sample when compared to that of the bare sample is attributed to the sacrificial nature of Al and Zn when the standard electrode potential of both the elements is more active than that of steel.

The corrosion current density (*I*_*corr*_) of Al-Zn coating is 7.19 µA/cm^2^ while that of bare steel is 20.10 µA/cm^2^. The coated sample exhibits 2.80 times lesser *I*_*corr*_ than bare steel in this type of an aggressive environment. Therefore, the corrosion rate of bare steel is higher than in the coated substrate. The corrosion rates of bare steel and Al-Zn pseudo alloy coating are calculated by inputting the *I*_*corr*_ value in equation 3 and correspond to 233.17 and 120.40 µm/year, respectively.

The potentiodynamic polarization results indicate that the coating exhibits superior corrosion protection efficiency, and thus other electrochemical studies with a longer duration of exposure including OCP measurements and electrochemical impedance spectroscopy (EIS) of coated samples are performed to elicit further details on the performance of coating in a saline environment and its mechanism.

#### Open circuit potential (OCP) measurements

**Figure 4:**
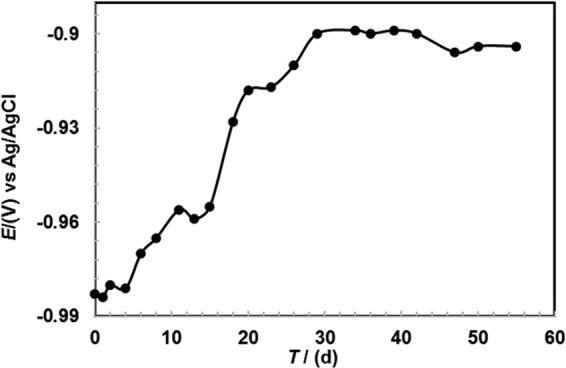
OCP measurements of Al-Zn coating applied by arc thermal spray process in 3.5 wt.% NaCl solution with exposure periods.

The OCP measurements of Al-Zn coating in 3.5 wt.% NaCl solution is performed with a number of exposure day/s (d) ranging to 55 d, and the results are shown in Fig. 4. The following trends are observed in the OCP values as a function of exposure time: (1) For initially up to 4 d of exposure, the OCP value moves towards the passive direction i.e., −0.984 V in 1 h to −0.981 V in 4 d vs. Ag/AgCl; (2) there is a positive shift of the OCP value from 6 d to 29 d (−0.970 V vs. Ag/AgCl for 6 d to −0.900 V vs. Ag/AgCl for 29 d); and (3) from 29 d onwards, the OCP value almost reaches a stabilized value of −0.904 V vs. Ag/AgCl.

The observance of the active potential during initial exposure period is attributed to the presence of defects/active sites on surface or composition of coating. The aforementioned observations are also noted by Y. Li wherein the addition of Al in galvanized coating induces the dissolution of Zn in sea water solution due to strong galvanic coupling or more electronegative potential than Al^14^. However, in present study, defects along with galvanic coupling are the governing factors towards the active dissolution of coating that resulted in more negative OCP. The ennobling in OCP after 15 to 29 d of exposure is attributed to the dissolution of coating although simultaneously the corrosion products block the active sites/defects of coating leading to the shift of the OCP towards a nobler direction. The phenomenon is potentially attributed to the deposition of corrosion products in coating defects that stifle the ingress of aggressive species towards the substrate^28,32^. From 29 to 55 d of exposure, the OCP of coating is almost stabilized (−0.904 V vs. Ag/AgCl), and it is potentially attributed to formation of compact, adherent, and uniform oxides/passive film that make the surface immune and function as a barrier against the penetration of the solution^52–57^.

The Al-Zn coating provides sacrificial protection to the steel throughout the exposure periods. It is commonly agreed that when coating exhibits OCP up to −0.8 V vs. saturated calomel electrode (SCE), it is considered as a good coating and provides sacrificial protection to the steel substrate in a sea water environment^58,59^. The aforementioned trend in OCP is observed for Al-Zn coating on steel substrate from −0.984 to −0.904 V vs. Ag/AgCl for 1 h to 55 d of exposure, respectively in the present study. The result indicates that the Al-Zn pseudo alloy coating by arc thermal spray process provides long term cathodic protection to the steel substrate in 3.5 wt.% NaCl solution. Therefore, it is concluded that the coating provides protection from corrosion over long term exposure to protect steel in a sea environment.

Thus, the corrosion process of Al-Zn coating in a saline environment is accomplished in three steps that include initiation of corrosion (from 1 h to 4 d), deposition of corrosion products on surface (from 4 to 29 d), and stabilization of corrosion phenomena (from 29 to 55 d).

#### EIS studies

**Figure 5:**
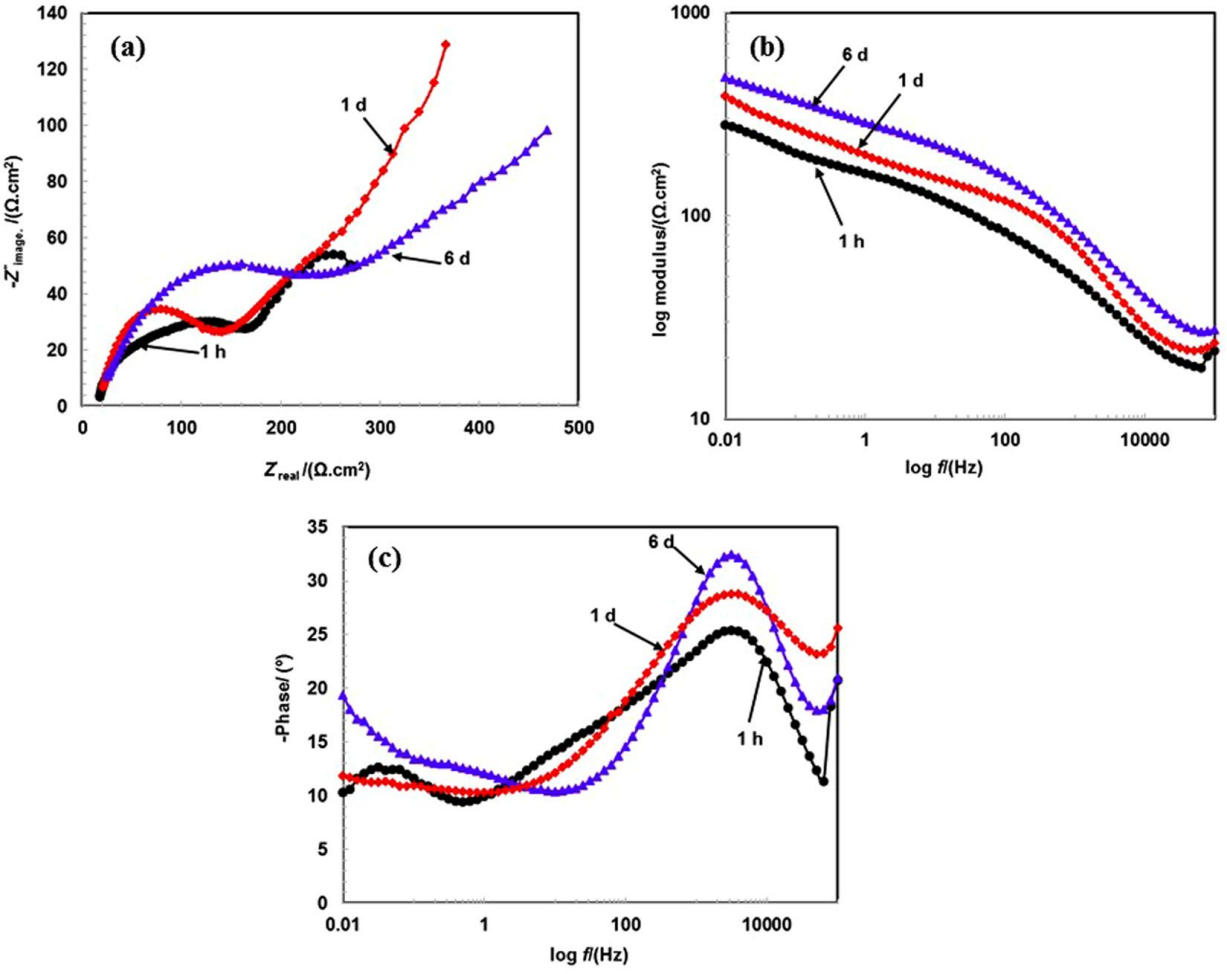
EIS studies (**a**) Nyquist (**b**) Bode log modulus frequency and (**c**) Bode phase frequency of Al-Zn pseudo alloy coating exposed in 3.5 wt.% NaCl solution after 1 h to 6 d of exposure.

The performance evaluation of coating is illustrated via an EIS study in 3.5 wt.% NaCl solution with passage of exposure periods. The EIS plots after 1 h to 6 d of exposure are shown in Fig. 5. The Nyquist plots exhibit the distinct feature of coating from 1 h to 6 d of exposure (Fig. 5a). After 1 h of exposure in 3.5 wt.% NaCl solution, the Nyquist plot exhibits smaller sizes compared to those at 1 and 6 d and this is attributed to the occurrence of corrosion phenomena at coating/solution interface. As shown in the FE-SEM image (Fig. 1), the coating deposited by the arc thermal spray process contains defects and pores on surface that can enhance the dissolution in 3.5 wt.% NaCl solution. Furthermore, after 1 h of exposure, the first half semi-circle loop at a higher studied frequency is observed due to the coating surface while a second incomplete semi-circle loop is related to the properties of oxide layer/solution interface during the corrosion process.

The nature of semi-circle loops changes when the exposure periods of coating in solution increase. After 1 to 6 d of exposure, it exhibits an identical tendency although the difference is observed only in the dimensions of the Nyquist diagram. After 6 d of exposure, the dimension of Nyquist plots at higher and lower studied frequencies exceed those at 1 h and 1 d. A possible reason for the trend is the increase in the exposure periods when the surface coverage area by corrosion products increases although the active surface area of coating simultaneously decreased, thereby decreasing the coating corrosion.

After 1 h of exposure, the impedance values of coating are extremely low at the low studied frequency (i.e., 0.01 Hz) as shown in Fig. 5b due to the presence of defects that enhance the dissolution^60^. However, when the exposure periods increase, the impedance values gradually increase, and this is potentially due to the transformation and deposition of corrosion products on coating surface. Initially, the coating exhibits defects/pores on surface that induce the ingress of aggressive ions and form corrosion products. When the exposure periods are extended, the deposition of corrosion products in the pores/defects of coating surface becomes prominent and significantly decreases the active surface area of coating, thereby increasing impedance values.

As shown in the Fig. 5b, impedance values at higher studied frequency (i.e., 100 kHz) increased with exposure periods, and the phenomenon is attributed to the deposition of corrosion products in pores of coating that causes resistance to the penetration of the solution towards the coating substrate.

The phase frequency Bode plots of coating from 1 h to 6 d of exposure in 3.5 wt.% NaCl solution are shown in Fig. 5c. The phase angle gradually shifts towards the higher side with exposure periods, and it is due to deposition of corrosion products on coating surface at higher studied frequency. The phase angle shifts from −25° to −32° at a higher studied frequency from 1 h to 6 d of exposure, and this is attributed due to the blocking of the active site by corrosion products on the coating surface.

The coating exposed for 1 h exhibits capacitive properties at a lower studied frequency due to defects on surface wherein shifting of maxima is achieved at −12° at 0.02 Hz. Conversely, after 6 d of exposure, it corresponds to approximately −19° at 0.01 Hz, and this is attributed due to the reaction that occurs at the passive film/solution interface wherein there is a lower chance for the ingress of solution through corrosion products. This is potentially because Zn is preferentially dissolved due to its more electronegative characteristics than Al, and thus the corrosion product blocks the active site of coating.

When the exposure periods increase from 13 to 55 d of exposure, the dimension and size of Nyquist plots increases in 3.5 wt.% NaCl solution when compared to that in prior studied periods. The gradual increments in the dimensions of Nyquist plots reveals the corrosion resistance properties of coating in 3.5 wt.% NaCl solution at longer durations of exposure. It is a well-established phenomenon that increases in the dimensions of Nyquist plots increases the corrosion resistance of coating and decreases the corrosion rate. Therefore, the aforementioned electrochemical hypothesis holds in this case when the corrosion rate of coating significantly decreases with exposure periods. Furthermore, it is observed that the corrosion process is controlled by corrosion products as opposed to the Al-Zn coating applied by the arc thermal spray process.

**Figure 6:**
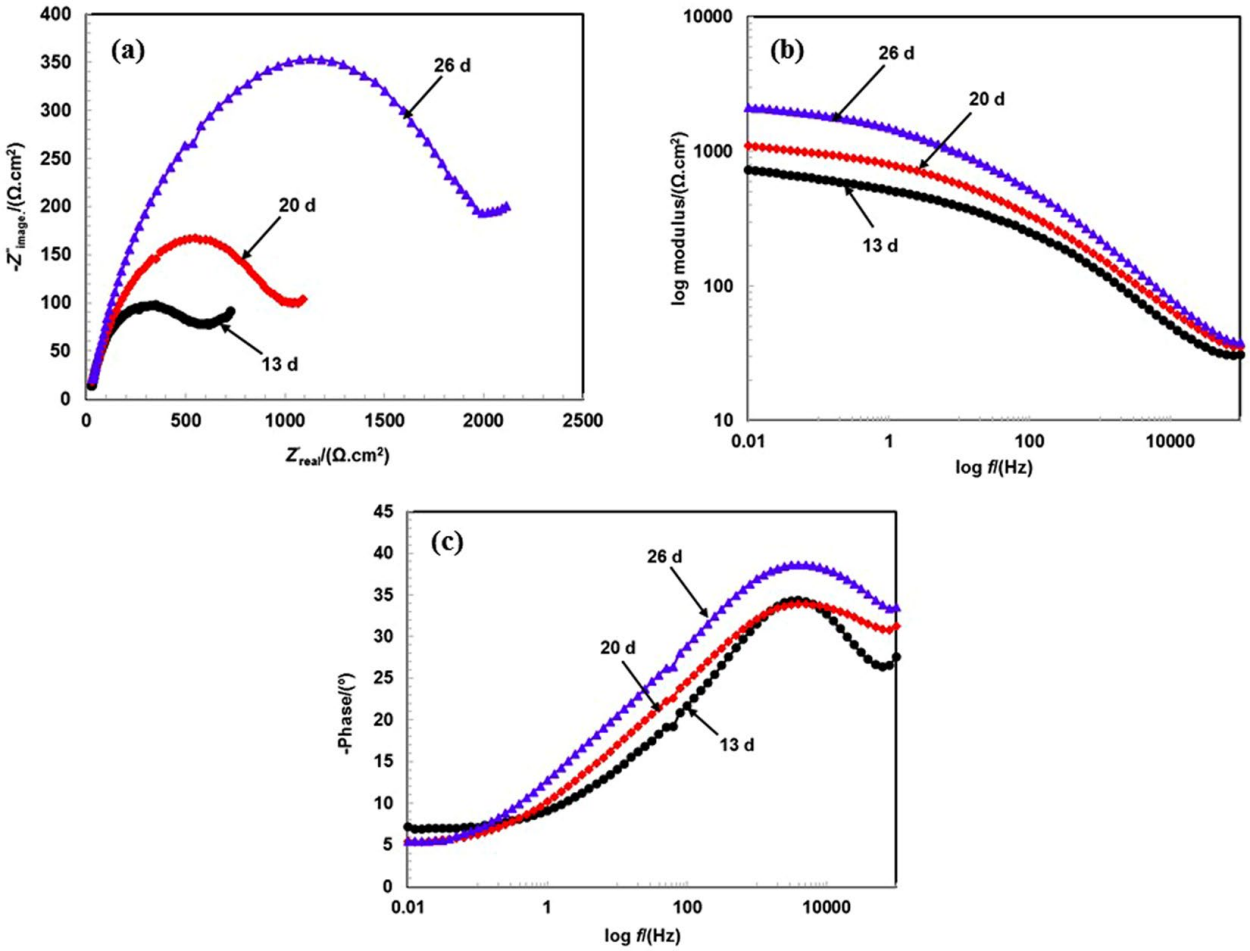
EIS studies (**a**) Nyquist (**b**) Bode log modulus frequency and (**c**) Bode phase frequency of Al-Zn pseudo alloy coating exposed in 3.5 wt.% NaCl solution after 13 to 26 d of exposure.

**Figure 7:**
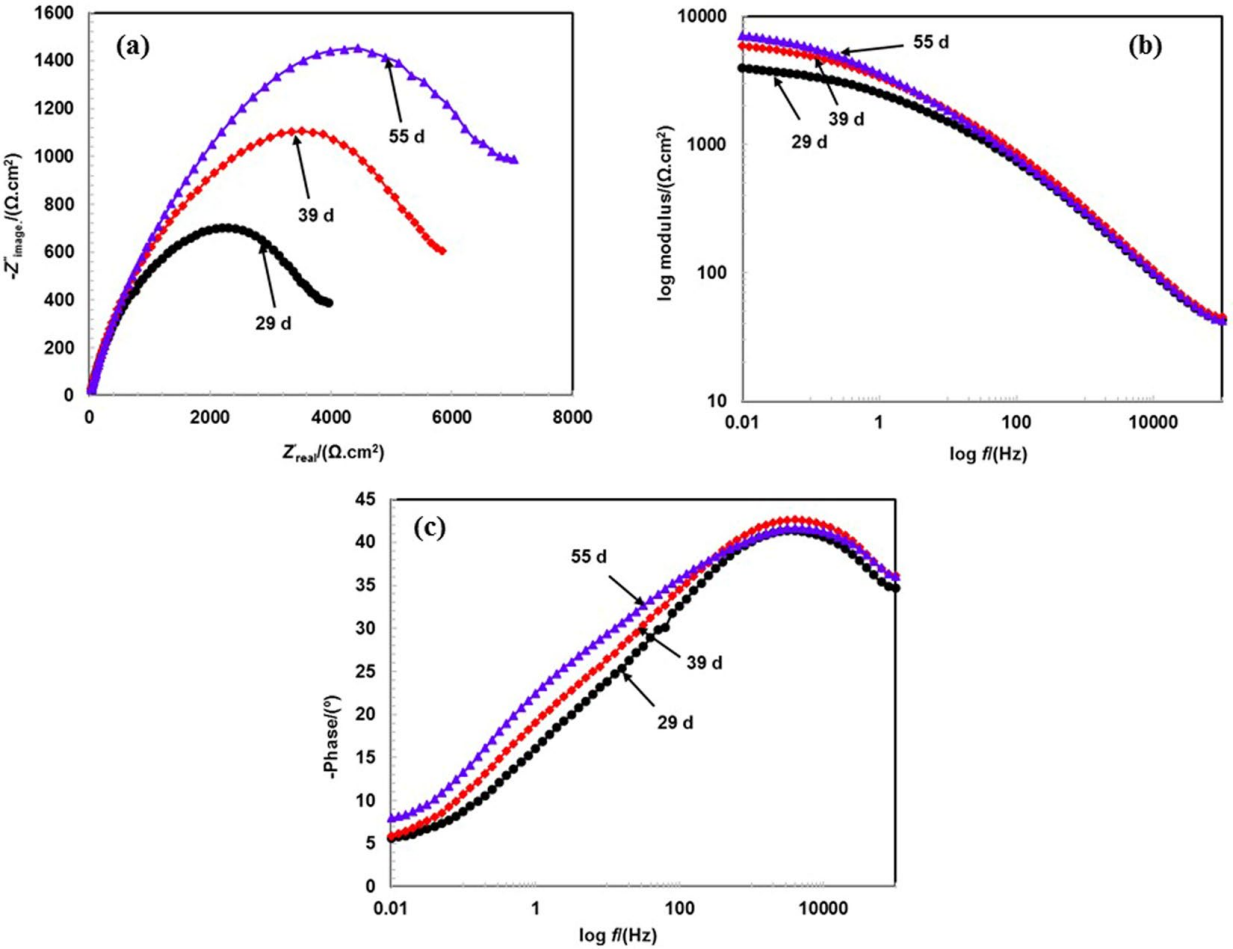
EIS studies (**a**) Nyquist (**b**) Bode log modulus frequency and (**c**) Bode phase frequency of Al-Zn pseudo alloy coating exposed in 3.5wt.% NaCl solution after 29 to 55 d of exposure.

The exposure periods contain one distinct half semi-circle loops in Nyquist plots at a higher studied frequency and a small tail at a lower studied frequency (Figs 6 and 7). The lower studied frequency plots are attributed to the deposition of corrosion products on the surface after the reaction of the coating with the solution at the coating/solution interface. This can be protective and contribute to reduce the active surface area of coating.

Furthermore, the thickening of corrosion products simultaneously occurs when the coating begins to dissolve after exposure in the solution. Initially, the corrosion products can be porous/defective, and thereby allow the ingress of aggressive ions of the solution towards the coating surface and result in the formation of corrosion products. When the duration of exposure periods increases, the thickness of corrosion products increases, and thus the dimensions of semi-circle loops increase and improve the corrosion resistance. The coating contains Al and Zn that are active metals for dissolution and begins to corrode after exposure to the NaCl solution.

As shown in Figs 6b and 7b, the impedance values at the lower studied frequency gradually increase with exposure periods, and this is attributed to the deposition of corrosion products on coating surface. The increase in impedance values with prolonged exposure periods indicate the formation of adherent, protective, and uniform corrosion products of Al and Zn.

The corrosion products block active surface areas, such as pores/cracks of coating, and enhance resistance to deterioration. However, a possibility exists that the corrosion products are uniformly deposited on surface and increase the polarization resistance. The impedance value at 0.01 Hz gradually increases from 13 to 26 d of exposure (Fig. 6b) although the value significantly increases (Fig. 7b) when compared to that in prior exposure periods after 29 d of exposure.

The result indicates that the deposition of corrosion products up to 26 d of exposure can contain a few defects although when the exposure periods increase beyond the period, the corrosion products can be uniformly deposited and enhance the polarization resistance. The impedance value at 100 kHz increases with exposure periods, and this indicates that the corrosion products are protective and less porous, thereby stifling the ingress of solution towards coating surface. The corrosion products work as a barrier at the solution/oxide interface. When the exposure periods increase, the impedance at 100 kHz is stabilized and almost identical from 29 to 55 d of exposure.

Phase-frequency Bode plots after 13 to 55 d of exposure are shown in Figs 6c and 7c. The phase angle maxima shift towards a higher angle from −34° to −43° after 13 to 55 d of exposure at the 5-kHz studied frequency. The shifting of the maxima at higher frequency indicates the dissolution of coating although it simultaneously shifts towards a higher angle. The result suggests that the coating is still under dissolution process at the coating/solution interface although the corrosion products deposit on the coating surface and causes capacitive property^46^. When the exposure periods increase from 13 to 55 d, both the medium and low frequency relaxation disappear, thereby indicating that corrosion products work as barriers themselves with respect to the deterioration of the coating^61,62^. Therefore, it is inferred that the Al-Zn coating worked as a self-healing coating wherein corrosion products heal the pores/defects of coating and enhance corrosion resistance properties at longer exposure durations.

**Figure 8:**
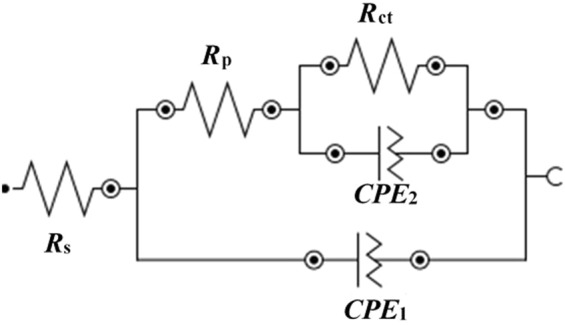
EEC of coating with exposure periods.

The electrical equivalent circuit (EEC) for the fitting of EIS data is shown in Fig. 8^4,5^. Based on the EIS plots, the coating is observed under active dissolution in 3.5 wt.% NaCl solution that contains different electrical parameters. These are described as *R*_*s*_, *R*_*p*_, *R*_*ct*_, and *CPE* that correspond to solution resistance, polarization resistance, charge transfer resistance, and constant phase elements, respectively. Two EEC are connected to each other. The *R*_*p*_ of first EEC is connected in series with the *R*_*ct*_ of the second EEC. The *R*_*ct*_ and *CPE*_2_ are parallel to each other. The first EEC reveals the properties of the coating caused by *R*_*p*_ while the second one is due to deposition of corrosion products caused by *R*_*ct*_. The *CPE* is due to the distribution of time constant that originates from two-dimensional roughness and inhomogeneity of the coating. The *CPE*_1_ and *CPE*_2_ in Fig. 8 correspond to the pseudo double layer capacitance and capacitance at lower studied frequencies, respectively.

An effective CPE coefficient (*Q*_*eff*_) is calculated by the imaginary impedance when *n* ≠ 1 in equation 4^63^ as follows:

4 $${Q}_{eff}=\,\sin (\frac{n\pi }{2})\frac{-1}{{Z}_{j}(f){(2\pi f)}^{n}}$$

where *n* denotes the *CPE* exponent, *Z*_*j*_ denotes the imaginary impedance, and *f* denotes the frequency. However, when *n* = 1 then *Q*_*eff*_ corresponds to capacitance (*C*_*eff*_), and equation 4 is expressed as:

5 $${Q}_{eff}={C}_{eff}=\frac{-1}{{Z}_{j}(f)(2\pi f)}$$

The blocking characteristics of coating between interfacial capacitance and the CPE coefficient (*Q*) are calculated by Brug’s equation^64^ and others^65,66^ as follows:

6 $${C}_{eff}={Q}^{1/n}{R}_{s}^{(1-n)/n}$$

**Figure 9:**
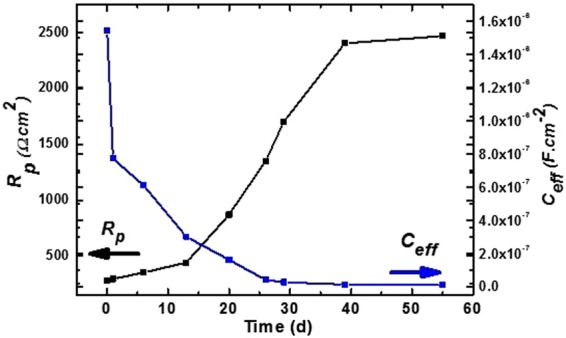
Plot between *Rp* and *C*_*eff*_ against time exposed to 3.5 wt.% NaCl solution.

The electrochemical parameters after fitting of EIS data in an appropriate EEC are shown in Table 2 and Fig. 9. As shown in the table, there are no differences in *R*_*s*_ (12.14 Ω.cm^2^ for 1 h to 14.79 Ω.cm^2^ for 55 d) with exposure periods. However, the *R*_*p*_ gradually increases to 13 d of exposure, and thus sudden improvement in its value is observed up to 29 d (Fig. 9). This emphasizes that the corrosion product becomes protective and adheres to the surface. The stabilization in *R*_*p*_ is observed after 29 d to 55 d of exposure, and this is attributed to the formation of stable film on the coating surface. However, the *C*_*eff*_ value drastically decreases up to 13 d of exposure thereafter gradual decrement although it is stabilized from 29 d to 55 d (Fig. 9). The result suggests that the corrosion product becomes homogenous, uniform, less defective, and adheres to the surface after 29 d of exposure in the 3.5 wt.% NaCl solution. The observation indicates that the active surface area decreases by the formation of corrosion products on the coating surface. The corrosion product causes capacitance and forms *C*_*eff*_. The *R*_*p*_ and *C*_*eff*_ are the parameters of coating that are directly related to the performance of materials. Furthermore, *Q*_1_ and *Q*_2_ gradually decrease with exposure periods, thereby indicating that coating and corrosion products become non-conducting, less defective, and exhibit less capacitive properties.

**Table 2:** Electrochemical parameters of Al-Zn coating were extracted from EIS plots after fitting of curves in suitable EEC in 3.5 wt.% NaCl with exposure periods.

Time	Electrochemical parameters					
	*CPE*_1_			*CPE*_2_		
	*R*_*s*_	*Q*_1_ (1 × 10^−4^)	*n*_1_	*R*_*ct*_	*Q*_2_ (1 × 10^−4^)	*n*_2_
	(Ω.cm^2^)	(Ω^−1^cm^−2^s^−n^)		(Ω.cm^2^)	(Ω^−1^cm^−2^s^−n^)	
1 h	12.14	4.94	0.47	306.22	39.70	0.40
1 d	12.67	3.11	0.48	397.97	35.76	0.40
6 d	12.84	2.76	0.48	528.98	33.08	0.41
13 d	13.46	1.92	0.48	600.05	32.35	0.42
20 d	13.77	1.24	0.49	668.09	31.61	0.42
26 d	13.94	0.63	0.49	993.63	6.43	0.47
29 d	14.20	0.33	0.52	2690.8	1.94	0.47
39 d	14.74	0.19	0.53	3887.5	1.24	0.49
55 d	14.79	0.17	0.53	6084.3	0.95	0.49

The *n*_1_ and *n*_2_ values consequently increase with exposure periods. However, the result indicates that the coating and corrosion products exhibit inhomogeneity in nature. The *R*_*ct*_ of coating significantly increases from 306.22 to 6084.30 Ω.cm^2^ from 1 h to 55 d of exposure, respectively. Given the presence of pores/defects on coating surface, it is primarily rough, and thus the *R*_*p*_ and *R*_*ct*_ decrease although the corrosion products are deposited on pores/defects and block the active surface area when deterioration commences. This is potentially because the corrosion products are uniform, adherent, and thick, and thus *R*_*p*_ and *R*_*ct*_ increase significantly with exposure periods in the 3.5 wt.% NaCl solution.

### Characterization of corrosion products formed on Al-Zn pseudo alloy coating surface after 55 d of exposure in 3.5 wt.% NaCl solution

**Figure 10:**
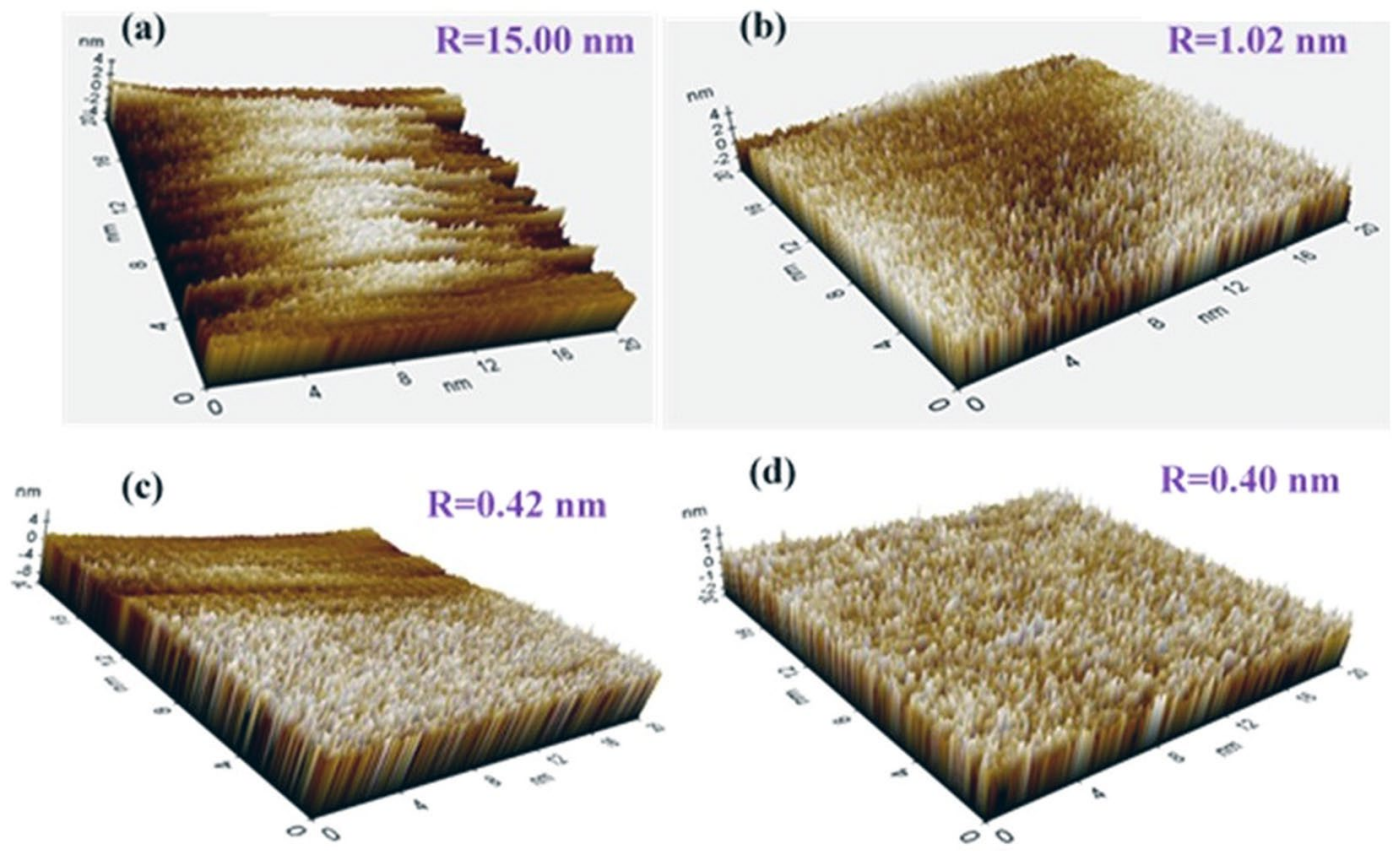
AFM results (**a**) Al-Zn coating (un-exposed) and after (**b)**1 d, (**c**) 29 d and (**d**) 55 d of exposure in 3.5 wt.% NaCl.

The comparison of Al-Zn (un-exposed) coating and corrosion products with different exposure periods in 3.5 wt.% NaCl solution is characterized via atomic force microscope (AFM), and the results are shown in Fig. 10. The 3D topography of un-exposed coating is shown in Fig. 10a, and it contains white deposition that is potentially due to a tiny oxide layer with a valley type orientation. The result indicates that the coating is defective, rough, and uneven, and this can allow penetration of the aggressive ions from atmosphere. The surface can cause active deterioration of coating. Conversely, it is observed that after exposure of the coating in solution, the topography of the coating surface changed when compared to that of an un-exposed coating. The deposition of corrosion products exhibits a few spikes on surface after 1 d of exposure (Fig. 10b) although the topography is uniform and exhibits regular orientation at prolonged duration of exposure (Fig. 10c,d). The dark area is observed in the AFM images (Fig. 10a) due to either attribution of surface morphology or defocusing. The coating and corrosion products are coarse while the selected surface area to collect the images is 20 nm × 20 nm, thereby making it possible to defocus the surface that results in the observation of the dark region. Given the bigger particle size, it is not possible to select an area that exceeds the aforementioned surface area, and thus we select an extremely low area to scan the surface of coating and corrosion products.

However, as shown in the Fig. 10d, after 55 d exposure of the coating in the solution, corrosion products are thick and uniformly deposited, and thus offers resistance to the penetration of the solution towards the coating surface. The average roughness (R) of the coating is calculated by using XEI-100 image processing software. The average roughness values for the coating in the un-exposed, 1 d of exposure, 29 d of exposure, and 55 d of exposure conditions correspond to 15, 1.02, 0.42, and 0.40 nm, respectively. The corrosion products/passive films regularly originate in the vertical direction with uniform orientation, and the distance between two uniform spikes/lattice spacing of corrosion products is minimal and dense. Therefore, the roughness at prolonged exposure periods significantly decrease when compared with that in the unexposed condition. Our results corroborate with those obtained in the study by Hussain *et al*. where they selected a scan range of 15 nm × 15 nm to calculate average lattice spacing^67^. Their results indicated that the average lattice spacing of passive film decreased with exposure periods of steel substrate in an alkaline solution. The results indicate that increases in the exposure periods decrease the roughness of coating. Extant studies report that decreases in the roughness causes the surface to be more corrosion resistant^30,68,69^.

**Figure 11:**
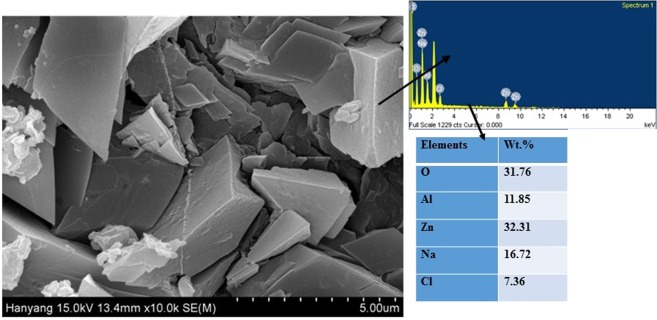
FE-SEM and EDS analysis of corrosion products after 55 d of exposure of Al-Zn coating in 3.5 wt.% NaCl solution.

The FE-SEM micrograph of corrosion products after 55 d of exposure in solution is shown in Fig. 11, and the corresponding EDS analysis are denoted by arrows on right side of the figure. The morphology of corrosion products is adherent and plate like, and they are uniformly distributed throughout the coating surface, thereby hindering the penetration of the solution towards the substrate. A few micro cracks are present that can allow penetration of the solution although the thick corrosion layer reduces the ingress of aggressive ions. Therefore, enhanced corrosion resistance is observed. The corrosion products particles are dilapidated with different sizes. The particles are rectangular, elongated, and exhibit increased size. The orientation of corrosion product particles is different and are recumbent on surface while others are flattened and vertically face the top surface. The corrosion product particle sizes range from 0.5 to 1.5 µm although the average size is 0.72 µm (±0.42 µm). The particle size is calculated by using ImageJ software.

Some amount of white deposition is observed in corrosion products, and this is potentially due to the presence of NaCl or oxides of Zn or Al. The test solution contains 3.5 wt.% NaCl that can be deposited on coating/corrosion products. The EDS analysis of corrosion products confirms the presence of O, Al, Zn, Na, and Cl. The O content in corrosion products is significant when compared with that of the coated sample. The O content is determined as 31.76 wt.%, and it is 10 times greater than as coated samples. It is due to formation of a few oxides/hydroxides during the corrosion process. Conversely, the amounts of Al (11.85 wt.%) and Zn (32.31 wt.%) in corrosion products are almost half of the coated sample. The decreased amount of the aforementioned metals in corrosion products are attributed either due to corrosion or extremely loosely bound oxides/hydroxides/chlorides of Al and Zn. The solution reacts with the main components (i.e., Al and Zn) of the coating and form a few corrosion products. Therefore, it is likely that the loosely bound oxides leach out in the solution or dissolve while adherent oxides/hydroxides adhere to the coating surface to fill/block the coating pores. There is a possibility of the formation of oxides/hydroxides/chloride of Al and Zn, such as Al_2_O_3_/Al(OH)_3_/AlCl_3_ or Zn(OH)_2_/ZnO/ZnCl_2_/Zn_5_(OH)_8_Cl_2_.H_2_O, and this is explained by Ahmido *et al*.^49^ and Zhang *et al*.^50^ Specifically, Al_2_O_3_/Al(OH)_3_/AlCl_3_ and Zn(OH)_2_/ZnO/ZnCl_2_ are mainly loosely bound oxides/hydroxides/chloride that easily leach out or dissolve in a solution while Zn_5_(OH)_8_Cl_2_.H_2_O (Simonkolleite) is sparingly soluble. Therefore, the chances for the formation of Simonkolleite in corrosion products is higher than that in others. In order to confirm the formation of Simonkolleite, we characterize the corrosion products via XRD and Raman spectroscopy. The details are explained in the subsequent paragraphs.

**Figure 12:**
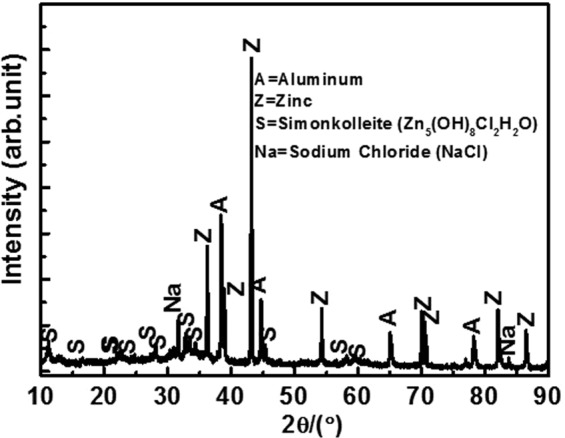
XRD of corrosion products formed on Al-Zn coating after 55 d of exposure in 3.5 wt.% NaCl solution.

The phase analysis of corrosion products was performed via XRD, and the results are shown in Fig. 12. The XRD analysis reveals the presence of Al, Zn, NaCl, and Zn_5_(OH)_8_Cl_2_.H_2_O (Simonkolleite). However, the XRD peak intensity of Al and Zn are lower than that in as coated samples (Fig. 2). The results reveal that the formation of another phase in the corrosion products is responsible for the reduction in peaks intensity. The JCPDS number for Al, Zn, NaCl, and Simonkolleite is obtained and correspond to 85–1327, 87-0713, 75-0306, and 07-0155, respectively. There is a possibility of the formation of another oxides/hydroxides or chlorides that can include Al_2_O_3_, Al(OH)_3_, AlCl_3_, ZnO, Zn(OH)_2_, and ZnCl_2_. The Al and Zn can react with NaCl solution and form these compounds. However, the compounds are unstable and soluble in water. Furthermore, the peaks are not obtained in XRD results, and this can be either due to extremely less amount or thin layer that is beyond the limit of XRD instrument. The formation of Simonkolleite is favorable during the corrosion of Zn or Zn/Al alloy or coating in NaCl containing environment^62^. The Simonkolleite is sparingly soluble in water, and most stable corrosion products of zinc are exposed to a chloride environment at neutral pH^70,71^. Given the insolubility of Simonkolleite in water, it accumulates in pores and cracks of surface that block the cathodic site and improve the corrosion resistance properties of the coating^71^.

The *V*_*f*_ of each phase is calculated by using the integrated surface area method. The *V*_*f*_ of Al, Zn, NaCl, and Simonkolleite correspond to 28.0, 47.27, 9.24, and 15.49%, respectively. The decrease in the *V*_*f(Zn)*_ of corrosion products reveals that Zn can more actively deteriorate in Al-Zn coating, and it is 1.45 times lower than that of un-exposed (68.50%) samples. This is potentially because the remaining Zn dissolves and forms its oxides/hydroxides/chlorides along with some amount of Simonkolleite. The amount of *V*_*f(Al)*_ is slightly less when compared to that of coated samples (31.50%) due to the extremely low dissolution, and there is a possibility of formation of Al(OH)_3_/Al_2_O_3_/AlCl_3,_ which are soluble in solution. Therefore, the phases are not detected by XRD. However, extant studies report that Simonkolleite is extremely stable, adherent, protective, and insoluble, thereby resulting in improved corrosion resistance properties of Al-Zn coating at longer durations of exposure in an NaCl containing solution^70,71^.

**Figure 13:**
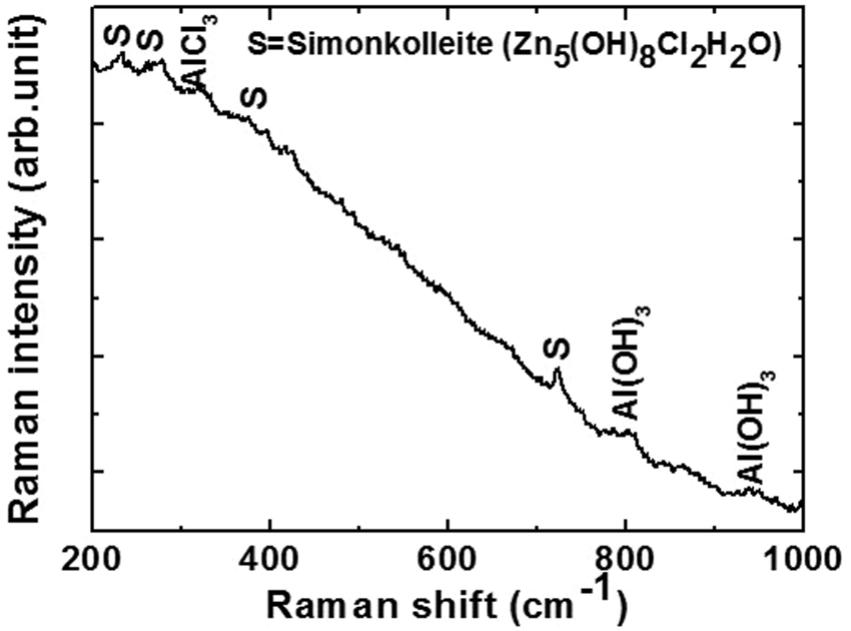
Raman spectra of corrosion products formed on Al-Zn coating after 55 d of exposure in 3.5 wt.% NaCl solution.

Raman spectra of corrosion products are shown in Fig. 13. The graph is plotted from 200 cm^−1^ to 1000 cm^−1^ due to increased noise, and no other peaks are observed at higher Raman shift. The corrosion products formed on the coating surface are uneven and nonhomogeneous. Therefore, the scattering of light on the surface was not uniform and results in the movement of the spectral base line (Fig. 13). The movement in Raman spectra is either due to sample preparation issue or low laser power used to collect the result as discussed by Hendra^72^. The corrosion products mainly contain Simonkolleite, AlCl_3_ and Al(OH)_3_, and they are detected in Raman spectra (Fig. 13). The Raman peaks at 226, 260, 400, and 725 cm^−1^ reveal the presence of Simonkolleite^59,73,74^ while 322 cm^−1^ corresponds to AlCl_3_ phase^75^ and 810 and 951 cm^−1^ correspond to the Al(OH)_3_ phase^76^. Although, AlCl_3_ and Al(OH)_3_ was not detected in XRD pattern, and it is potentially due to the extremely thin layer and low amounts. The presence of AlCl_3_ and Al(OH)_3_ are detected by Raman spectroscopy in corrosion products due to reaction of Al with the NaCl containing solution. They are easily detached from the coating surface and dissolved in solution and provide strong evidence, thereby corroborating the EDS, XRD, and our hypothesis. Specifically, Al, Zn, and NaCl are non-sensitive for Raman spectroscopy, and thus they are not detected by the instrument.

### Corrosion protection mechanism of Al-Zn pseudo alloy coating in 3.5 wt.% NaCl solution with exposure periods

**Figure 14:**
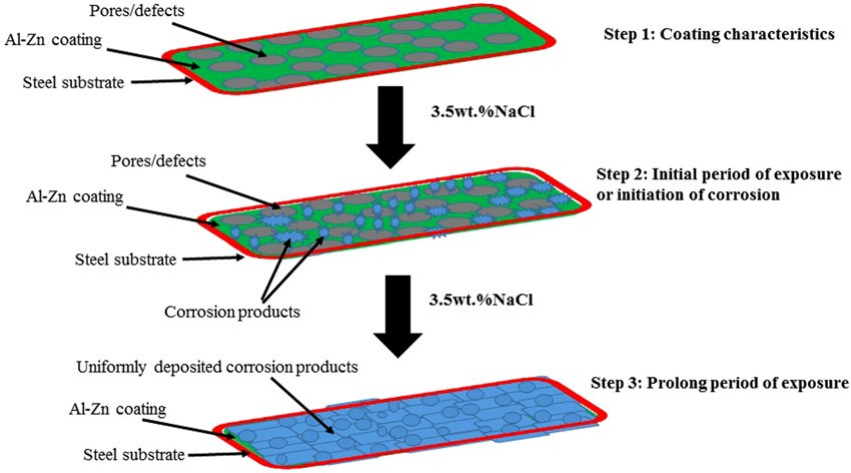
Schematic presentation of corrosion process of Al-Zn coating applied by arc thermal spray process in 3.5 wt.% NaCl solution with exposure periods.

The corrosion mechanism and protective properties of Al-Zn coating are explained by the schematic (Fig. 14). The process consists of three different steps. The first step involves explaining the process of coating wherein the Al-Zn coating is applied by arc thermal spray process on steel substrate when it exhibits several pores/defects on the surface. The defects allow the penetration of the aggressive ions during exposure in the solution, and this causes or initiates the corrosion process. The second step involves the initial exposure of coating in 3.5 wt.% NaCl solution where the initiation of the corrosion process is accelerated. During the process, the deposited coating begins to dissolve and form corrosion products. Initially, the amount of corrosion products is extremely less with porous morphology that can allow for the ingress of the solution through it. The duration can range to 26 d of exposure.

Following the occurrence of proper corrosion, the thickening and nature of corrosion products are dominant over deterioration. The process is explained in the third step. The dominant nature of corrosion products deposition over the deterioration process of Al-Zn coating is observed from 29 d to 55 d of exposure. The corrosion products are thick, uniform, adherent, and protective. Therefore, the enhanced corrosion resistance properties of the Al-Zn pseudo alloy coating in 3.5 wt.% NaCl solution are observed with its prolonged exposure.

## Conclusions

The following conclusion are drawn from the present study:

1. The deposited Al-Zn pseudo alloy coating through arc thermal spray process exhibited defects/cracks and inflight particles as observed by FE-SEM due to the limitation of instruments used to deposit the coating.
2. The results of the present study indicate that the primary coating exhibited deterioration. However, when the proper reaction of coating with solution or aggressive ions occurred, it formed protective, adherent, insoluble, uniform, thick, and protective corrosion products that significantly reduced the corrosion rate of coating at longer durations of exposure.
3. The Al-Zn coating exhibited active *E*_*corr*_ and OCP at approximately −0.9 V vs. Ag/AgCl throughout all the exposure periods, thereby indicating that sacrificial protection is provided by the coating to the steel.
4. The EIS studies verified that *R*_*p*_ and *R*_*ct*_ are initially low although these values increase at longer duration of exposure in 3.5 wt.% NaCl solution, thereby resulting in a reduced corrosion rate.
5. The corrosion products blocked the porosity of coating, thereby resulting in higher *R*_*p*_ and *R*_*ct*_.
6. The reduction in the roughness of corrosion products with exposure periods was confirmed by AFM technique, and this supported the hypothesis for the enhanced corrosion resistance of Al-Zn coating at prolonged exposure periods in a saline solution.
7. The XRD and Raman spectroscopy results confirmed the formation of Simonkolleite as the corrosion product of Al-Zn coating that is adherent, protective, and sparingly soluble in water.

## Materials and Methods

### Process of coating

In the study, Al-Zn alloy coating was deposited on 0.08 m × 0.06 m × 0.001 m dimension of mild steel plate containing C = 0.20, Mn = 0.95, Si = 0.26, P = 0.02, S = 0.01, Cu = 0.02, Cr = 0.04, Ni = 0.03, Fe = balance in wt.% by arc thermal spraying process. Prior to depositing the coating, the steel substrate was properly pickled with 10 v/v % HCl for 10 min to remove the contaminant, oil, grease, and oxides on surface and then thoroughly washed with distilled water and dried at 25 °C (±1 °C). After drying, the steel plate was grit-blasted with 0.7 and 0.8 mm steel balls by using a pressure machine at 0.7 MPa to make the surface rough for proper mechanical bonding with the deposition of a metallic coating.

The arc thermal spray process included different parts such as spray gun containing nozzle with consumable wires, roller, power supply, and compressed air. Specifically, 1.6-mm diameter mesh wires of commercially pure Al and Zn (purity 99.95 wt.%) were used to deposit the coating on steel substrate, and they were fixed in the spray gun. With the help of a roller, two dissimilar twin wires of Al and Zn metal were simultaneously used to deposit the coating via an arc thermal spray process. The twin wires came out from the nozzle via roller and were fixed in a circular slit as shown in Fig. S1 (Supplementary Fig. 1)^77^. Subsequently, both wires met at the arcing point and began to melt due to oppositely charged power supply when hot and compressed air exerted pressure over the melted metal particles and propelled them towards the steel substrate^78–80^. The melted metals droplets began to spread over the substrate to be coated and formed the coating^21^. Given the sudden cooling at atmospheric temperature, the resulting thick coating was formed with pores/defects. The bonding between substrate and deposited coating was high, intact, and mechanical as opposed to intermetallic due to the kinetic energy of sprayed particles and increased affinity of metals^24^. The deposition of Al-Zn coating on steel surface was accomplished via keeping the sample at a distance of 0.2 m from the spray gun at an air pressure of 6 bars. In the spraying process, voltage and current were maintained at 30 V and 200 mA, respectively^81–83^.

The density, atomic weight, and melting point of both metal wires were different and played a major role in the composition and morphology of deposited coatings. The Al metal exhibited lower density and higher melting point than Zn, and thus different concentrations of metals were obtained after the deposition of the coating. During the spraying, the Al particles were suspended in the atmosphere while Zn melted early and preferably deposited and adhered to the base substrate and formed the coating. Therefore, it formed a pseudo Al-Zn alloy coating as opposed to a pure metallic coating^84,85^.

After the deposition of the coating, the thickness was measured via a non-destructive technique by using Elcometer456 at three different locations.

The adhesion test of Al-Zn pseudo alloy coating deposited on steel surface was characterized via the pull off test. The pull off test is considered as the adhesion measurement of coating, and it was performed according to KS F4716^35^ standard.

### Electrochemical studies

The electrochemical studies of coating were performed in 3.5 wt.% NaCl solution. The solution was prepared by dissolving analytical grade of NaCl in double distilled water. Prior to commencing the experiments, the coating was kept in 3.5 wt.% NaCl solution for 1 h to stabilize the potential with potentiostat. The studies were performed by three electrode systems where coating acted as a working electrode, platinum wire acted as a counter electrode, and silver-silver chloride (Ag/AgCl) acted as the reference electrode (Fig. S2)^77^. The sample holder area of working electrode was 7.8 × 10^−5^ m^2^, and it was fixed for all samples.

Electrochemical impedance spectroscopy (EIS) studies were performed at open circuit potential (OCP) with an amplitude of 10 mV sinusoidal voltage by changing the frequency from 100 kHz to 0.01 Hz. Potentiodynamic polarization studies were performed at a scan rate of 1 mV/s from −0.4 V to +0.8 V vs. Ag/AgCl^86^. The potentiostat corresponded to VersaSTAT (Princeton Applied Research, Oak Ridge, TN, USA), and data analysis was performed by Metrohm Autolab Nova 1.10 software by fitting the experimental data in a constant phase element (CPE) model. All electrochemical studies were performed in triplicate at 25 °C (±1 °C) to obtain consistency in results.

### Characterization of coating and corrosion products

The top surface morphological analysis of Al-Zn pseudo alloy coating and corrosion products were assessed by field-emission scanning electron microscopy (FE-SEM, Philips XL 30, USA) operated at 15 kV, and it was equipped with an energy dispersive X-ray spectrometer (EDS) for elemental analysis. A thin platinum coating was applied prior to obtaining the FE-SEM images of coating and corrosion products that increased the conductivity and avoided the charging effect.

In order to examine the topography of coating and corrosion products, an atomic force microscope (AFM) was used (Park, XE-100) by keeping the samples 12 µm away from the working distance at Z-scanner. The scan range was 20 nm × 20 nm at the XY scanner via the non-contact mode. The scanner was decoupled with the X, Y, and Z axes. The analysis of AFM results was performed via XEI-100 image processing software.

In the study, X-ray diffraction (XRD, Philips X’Pert-MPD) studies of coating and corrosion product were performed with a Cu Kα radiation (λ = 1.54059 Å) generated at 40 kV and 100 mA. The sweeping scan ranged from 10 to 90° with a scan rate of 0.5°/min to collect the XRD data. The obtained peak identification was performed by comparing it with that of the Joint Committee on Powder Diffraction Standards (JCPDS) files.

The nature of corrosion products formed on the Al-Zn pseudo alloy coating surface after 55 d of exposure in 3.5 wt.% NaCl was characterized via Raman spectroscopy (Renishaw RM 1000) by using an Al-Ga-As diode laser beam with a wavelength of 758 nm.

## Supplementary information

Corrosion mechanism and kinetics of Al-Zn coating deposited by arc thermal spraying process in saline solution at prolong exposure periods
