# Supplementary material for: Corrosion mechanism and kinetics of Al-Zn coating deposited by arc thermal spraying process in saline solution at prolong exposure periods
Source: Sci Rep. 2019 Mar 4;9:3399. doi: 10.1038/s41598-019-39943-3 (PMC6399354; doi:10.1038/s41598-019-39943-3)
Supplement: Supplementary file 1 — Corrosion mechanism and kinetics of Al-Zn coating deposited by arc thermal spraying process in saline solution at prolong exposure periods [file 41598_2019_39943_MOESM1_ESM.pdf]

Corrosion mechanism and kinetics of Al-Zn coating deposited by arc thermal spraying process in saline solution at prolong exposure periods

Han-Seung Lee [1], Jitendra Kumar Singh [1, *], Mohamed A. Ismail [2], Chinmoy Bhattacharya [3], Asiful H. Seikh[4, *], Nabeel Alharthi [5] and Raja Rizwan Hussain [6]

[1] Department of Architectural Engineering, Hanyang University, 1271 Sa 3-dong, Sangrok-gu, Ansan 15588, Korea;

[2] Department of Civil Engineering, Miami College of Henan University, Jinming Avenue No.1 Kaifeng, Henan 475001, China.

[3] Department of Chemistry, Indian Institute of Engineering Science and Technology (IIEST), Shibpur, Howrah 711 103, West Bengal, India

[4] Centre of Excellence for Research in Engineering Materials, King Saud University, P.O. Box 800, Riyadh 11421, Saudi Arabia

[5] Mechanical Engineering Department, King Saud University, P.O. Box 800, Riyadh 11421, Saudi Arabia

[6] College of Engineering, Department of Civil Engineering, King Saud University, P.O. Box - 800, Riyadh 11421, Saudi Arabia

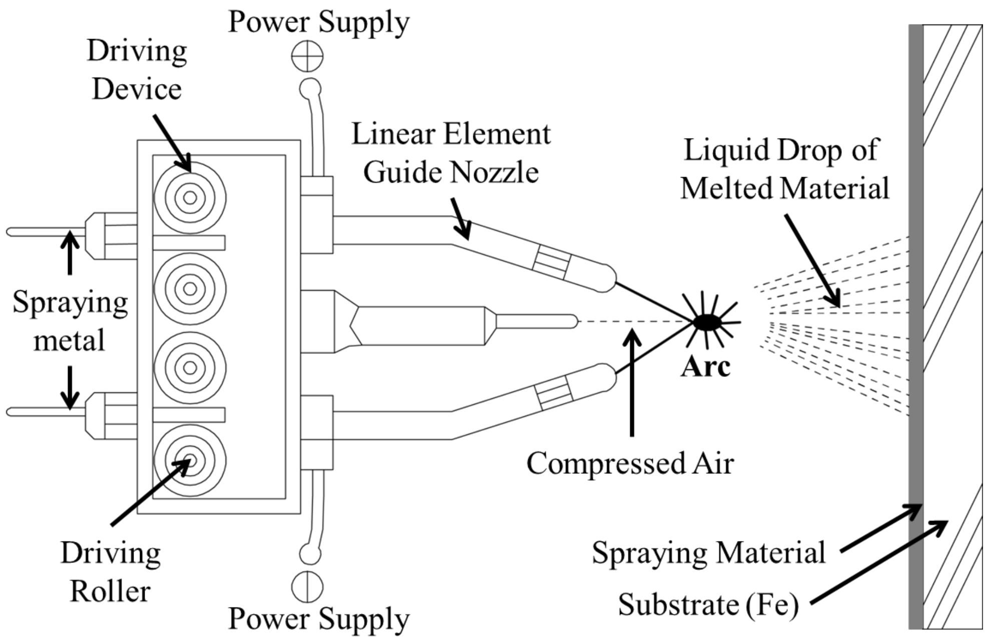

Figure S1. Schematic of arc thermal praying process[77].

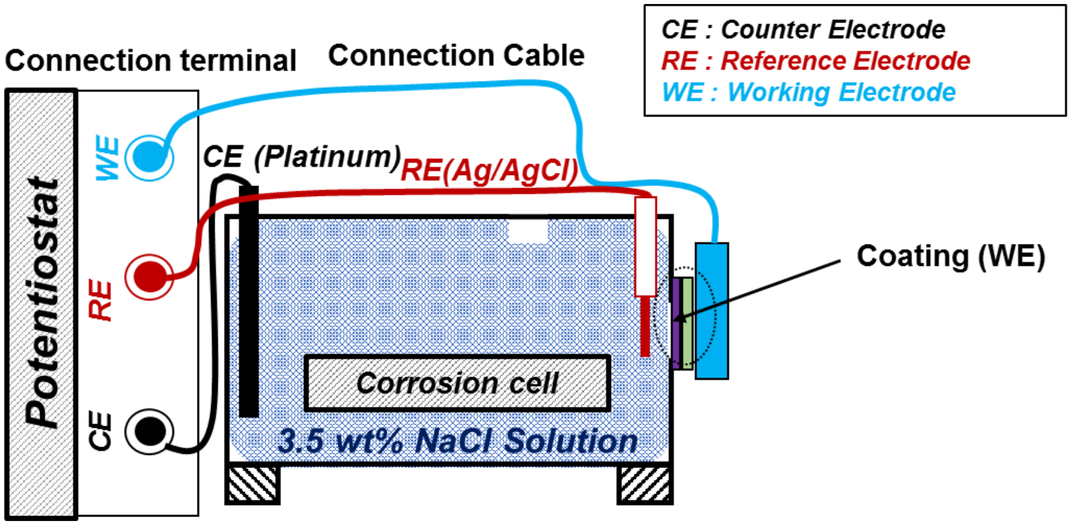

Figure S2. Schematic of electrochemical set up[77].
